# The “Ideal Birth”: The Occurrence of Severe Perineal Lacerations, Related Factors and the Possibility of Identifying Patients at Higher Risk

**DOI:** 10.3390/healthcare12242584

**Published:** 2024-12-22

**Authors:** Carmen Imma Aquino, Alessia Tivano, Francesca Della Sala, Sofia Colagiorgio, Lucia Scalisi, Tewobista Ewnetu Alemu, Lorenza Scotti, Elisabetta Tarrano, Valentino Remorgida, Daniela Surico

**Affiliations:** 1 Department of Translational Medicine, Università del Piemonte Orientale, Gynecology and Obstetrics, “Maggiore della Carità” Hospital, 28100 Novara, Italy; alessia.tivano@gmail.com (A.T.); francescadellasala@gmail.com (F.D.S.); sofiacolagiorgio@libero.it (S.C.); luciascalisi@hotmail.it (L.S.); elisabetta.tarrano@maggioreosp.novara.it (E.T.); valentino.remorgida@uniupo.it (V.R.); 2 Department of Translational Medicine, Unit of Medical Statistics, Università del Piemonte Orientale, 28100 Novara, Italy; 20053539@studenti.uniupo.it (T.E.A.); lorenza.scotti@uniupo.it (L.S.)

**Keywords:** severe perineal lacerations, OASIS, fecal and urinary incontinence

## Abstract

Background: Obstetric lesions of the anal sphincter (OASIS) are tears intersecting the structure of the anus after vaginal delivery. Our aim is to provide data on the incidence of OASIS and investigate potentially connected risk factors. Methods: This is a retrospective analysis of 464 parturient patients admitted to the AOU Maggiore della Carità, Novara (Italy), in the last ten years (2013–2023), comparing 116 cases (with OASIS) versus 348 controls (with no OASIS). Results: The incidence of OASIS was 1.1%. Among the significant risk factors associated with the risk of severe perineal laceration in our sample, we observed nulliparity, previous caesarean sections, assisted reproduction technology, kilos gained during pregnancy, induced delivery, the use of oxytocin for augmentation, epidural analgesia, delivery after 40 weeks of gestation, position at delivery, the duration of labor, the application of a vacuum cup, newborn weight and head circumference. Conclusions: It was a challenge to find data on OASIS and on more preventable and modifiable risk factors. Beyond the improvement of the corresponding diagnostic and therapeutic tools, a new aim could be to stratify women giving birth based on possible risk factors.

## 1. Introduction

Severe perineal tears, referred to as third- and fourth-degree perineal lacerations, affect 3.1% of vaginal deliveries, greatly impacting quality of life [1]. Nowadays, they are known as obstetric anal sphincter injuries (OASIS) and represent the most common cause of anal incontinence (fecal and flatus) in women of reproductive age [1]. Their effects on women’s health and mental well-being can be detrimental. Thus, an accurate diagnosis and appropriate management are crucial, and these aspects should be essential parts of obstetric training. Depending on the anatomical structures involved, OASIS fall into two categories: third-degree lacerations, which involve the anal sphincter complex, and fourth-degree lacerations, which extend to the rectal mucosa. Note that the third-degree lacerations are subclassified by the RCOG into three subgroups: third-degree A, involving less than 50% of the external anal sphincter; third-degree B, involving more than 50% of the external anal sphincter; and third-degree C, in which the internal anal sphincter is also injured [2,3]. Various factors can increase the chances of experiencing severe perineal lacerations; those most reported in the literature are increased birthweight, instrumental delivery, fetal occiput posterior position, and the duration of the second stage [3,4,5].

Identifying the risk factors for OASIS is necessary for patients’ health and may help future researchers to enhance diagnostic accuracy and target women at higher risk, addressing the modifiable conditions and experimenting with some preventive measures. The aims of our study are to deepen the information on the possible clinical–anamnestic, intra-partum, and neonatal factors correlated with high-grade perineal lacerations and define the incidence of OASIS, considering the lack of data in this regard.

## 2. Materials and Methods

A single-center case-control study was conducted at the Department of Gynecology and Obstetrics of the Maggiore della Carità Hospital in Novara (Italy). The study population consisted of women who gave birth at our center between 1 January 2013 and 1 July 2023.

The data were collected by consulting the Childbirth Certificates of Assistance database (CEDAP) and computerized medical records with the patients’ consent, which was given at the time of hospitalization. The following inclusion criteria were applied to make the sample relevant to the aim of this study. Women were included in this study if they (i) were aged ≥ 18 years, (ii) had a single pregnancy, and (iii) provided consent to participate in the study. Patients who did not give their consent to participate and/or gave birth by caesarean section were excluded. All cases involved women who reported high-grade lacerations (OASIS) at delivery. For each case, three controls of the same mean age and year of delivery were randomly selected among women who completed delivery without lacerations or with episiotomy, abrasions, or laceration of lower grades (1st or 2nd degree).

Several maternal anamnestic and obstetric factors related to the current pregnancy and factors related to delivery and neonatal characteristics were retrieved from CEDAP as potential predictors of OASIS. Specifically, the following information was considered: age, ethnicity, smoking status, use of medically assisted reproduction technology, parity, previous miscarriages, previous caesarean sections, pre-gravidic body mass index (BMI), previous pathologies, gestational pathologies, diabetes, weight gain during pregnancy, perineal massage, gestational age, labor induction, labor augmentation, position during delivery, instrumental delivery, duration of the active and expulsive phases, type of laceration, use of mediolateral episiotomy, epidural analgesia, shoulder dystocia and experience of the midwife who attended the delivery, birth weight, occiput position of the presented fetal part, and head circumference measurements.

The sample size was calculated assuming a first-type error of 5%, a power of 80%, and a risk factor prevalence of 0.43 among controls, observing a minimum-detectable OR of 1.32.

This study was authorized by the local Ethics Committee under protocol no. 753/CE, file no. CE022/2023.

### Statistical Analysis

The main characteristics of women included in this study were summarized using descriptive statistics; in particular, categorical variables were presented as absolute frequencies and percentages, and numerical variables were given as means and standard deviations (SDs) or medians and interquartile ranges (Q1–Q3), if not normally distributed according to the Shapiro–Wilk test. The Chi-square test or Fisher exact test was used to test for the presence of associations between categorical variables and the presence of OASIS, and a t-test or Mann–Whitney test was conducted to test for a link between numerical variables and the presence of OASIS. Univariate Poisson regression models with robust variance were used to estimate relative risks (RRs) and corresponding 95% confidence intervals (95% CIs) to assess the association between selected clinical variables and the risk of vaginal tears. Type 1 error was set to 0.05, and all analyses were performed using SAS 9.4 (SAS Institute, Cary, NC, US).

## 3. Results

The sample consisted of a total of 464 women, with 116 cases and 348 controls. In this study, we analyzed the incidence of OASIS in our hospital, finding a general incidence of 1.1%. Among the controls, 82.8% had no lacerations, and 10.3% and 6.9% had lacerations of the first and second grades, respectively; among cases, 95.7% had severe lacerations of the third grade (77.6% had type 3A, 13.8% had type 3B, and 4.3% had type 3C), and the remaining 4.3% had lacerations of the fourth grade. Descriptive statistics of maternal anamnestic factors, obstetric factors related to the current pregnancy, factors related to delivery, and neonatal characteristics overall and segregated by case or control status are reported in Table 1, Table 2 and Table 3. Most women (341) were aged <35, while the remaining 123 were over 35 years of age at the time of birth. The average BMI was 23.7 kg/m^2^ (SD of 4.6), and maternal ethnicity was predominantly Caucasian (72.0%). In total, 2.4% of the sample consisted of smokers. Overall, 52% (242 women) were multiparous patients, while 48% (222 women) were nulliparous, and 1.7% used medically assisted reproduction. The average fetal birth weight was 3,285 g (SD 495); the average head circumference at birth was 33.8 cm (SD 1.6). The median gestational age at birth was 278 days (Q1–Q3 272–283 days).

Data about comorbidities were also collected and then classified into: thyroid disorders, eating disturbs, hypertension, viral diseases, gestational cholestasis, coagulation deficits, anemia, risk of preterm birth, oligohydramnios, fetal growth restriction, preeclampsia, liver diseases, cardiovascular pathologies, antiphospholipid syndrome, nephrological diseases, and other conditions. However, considering the few pathological pregnancies in our sample, the patients were only classified as being physiological/pathological in the analysis (83.5% vs. 16.5%). A specific investigation was also conducted on the diabetic patients, who constituted a very small part of the study sample (10.3%). Among the diabetic women, 97.9% had gestational diabetes, and the remaining 2.1% were affected by this disease before pregnancy. According to the results of the statistical tests, nulliparity, the number of previous miscarriages and previous caesarean sections, use of assisted reproduction technology, kilos gained during pregnancy, induced delivery, the use of oxytocin as augmentation, epidural analgesia, delivery after 40 weeks of gestation, the position at delivery, the duration of labor, the application of a vacuum cup, and newborn weight and head circumference seemed to be associated with the risk of severe perineal laceration in our sample. Table 4 reports the RRs and corresponding 95% CIs used to evaluate the strength of the association between maternal, pregnancy, intra-partum, and newborn variables and the risk of OASIS.

Regarding the maternal variables, previous caesarian sections, undergoing assisted-reproduction pregnancy, and nulliparity increase the risk of high-grade tears 2.3-, 3.1-, and 7.3-fold, respectively. Also, weight gain during pregnancy turned out to be a risk factor for OASIS but only when the amount of weight gained was >15 kg, when compared to <10 kg (RR1.68, 95% CI 1.04–2.72). Concerning delivery, induction and labor augmentations were risk factors for severe lacerations since women who underwent induction had a 53% increased risk of OASIS compared to women who did not need induction and as women who needed labor augmentation had a 2.3-fold-higher risk of lacerations compared to women who did not need it. Further risk factors of severe lacerations were post-term pregnancy, the use of epidural analgesia, and the application of an obstetric suction cup. Regarding the characteristics of newborns, greater birthweight and head circumference increased the risk of OASIS by 10% and 35%, respectively.

The results of the multivariate model confirm that the application of an obstetric suction cup, greater head circumference, gestational age, and the duration of the first and second stages of labor increased the risk of OASIS. Conversely, women who underwent episiotomy had an approximately 70% lower risk of severe lacerations compared to women who did not need to undergo this procedure.

## 4. Discussion

This retrospective study highlighted some interesting clinical aspects that are useful in daily practice, with statistically significant results supported by the literature [4].

We identified several risk factors for the occurrence of OASIS: previous caesarean section, the use of medically assisted procreation techniques, maternal weight gain (greater than 15 kg), primiparity, childbirth beyond 40 weeks of gestation, analgesia, the position at delivery, post-term gestational age at delivery, the application of an obstetric suction cup, greater fetal birthweight and head circumference, and a greater duration of the first (dilating phase) and second stages of labor (expulsive phase).

In our results, a previous cesarean section before a spontaneous birth was associated with a significantly increased risk of OASIS. A systematic review published in July 2023 supported the association between TOLAC (trial of labor after caesarean) and severe perineal lacerations (OR: 1.46, 95% CI 1.12–1.92) [5]. Medical induction of labor is also associated with an increased risk of severe tears; an example is the use of oxytocin: for induction or acceleration of labor, this seems to increase the risk of OASIS. These latest results are also in line with the association detected over the years [4].

Participation in birthing classes is a potential tool that helps prevent OASIS. Despite the composition of our control group, most of whom consisted of multiparous women who had not participated in preparatory courses, we remain supporters of this preparatory step. Birth support courses are universally recognized as fundamental not only during the first gestation but also in subsequent pregnancies. Guidelines for the physiological management of pregnancies recommend “offering information and opportunities for discussion about topics deemed relevant by women [6,7], supported by printed material or otherwise information support”.

The studied risk factors related to the delivery room include the use of childbirth analgesia, fetal birth weight, the duration of labor, the position at delivery, and fetal head dimensions. Based on our data, women who receive analgesia are at a higher risk of developing OASIS. 

Furthermore, for every 100 g increase in fetal birth weight, the risk of OASIS increases by 10%. Similarly, each further increase of 10 min in the duration of the expulsive period during labor is associated with a 12% increase in the risk of experiencing severe perineal tears. To date, few studies have confirmed these correlations, particularly between birthweight and fetal head circumference and the percentage increase in the risk of OASIS [8,9].

While collecting our data, we also examined whether midwifery work experience could influence the perineal outcomes. In particular, we divided work experience into more than or less than five years. The scientific literature is not unequivocal on this subdivision, which is well-established among other healthcare professionals [10]. Recently, in our obstetric department, we observed a significant turnover of healthcare personnel, with a considerable increase in midwives with less than five years of work experience.

In addition to these findings, another objective of the present study was the evaluation of the incidence of OASIS at our center, considering our hospital as a reference for Europe in general: our center is in fact a medium-sized and highly active university hospital (with 2000 deliveries per year). The computerization of medical records starting in 2017 has greatly simplified the collection of data.

It is difficult to find a precise incidence in the literature for some Western centers, particularly Italian hospitals. A slight increase in our OASIS cases occurred in the 2020–2021 period, followed by a return to the mean in 2022. It is essential to mention the possible influence of the COVID-19 pandemic that occurred in this period. It is plausible that the restrictions imposed, and the psychological, physical, and pathological changes induced by this disease may have had an impact on the course of pregnancy and consequently childbirth, contributing to the increase in cases [11,12]. Furthermore, it is also important to consider the possibility of an increase in dystocic, induced, and/or vaginal operative deliveries, which may have contributed to this phenomenon [3].

On average, the global incidence of OASIS at our hospital in the last few years has been stable, amounting to around 1.1%. Cases are always related to the number of births, which shows a trend with respect to the number of national data. The current availability of targeted studies on this topic in the literature is limited, requiring more research to be conducted.

The strength and novelty of our results mainly lie in the preventable and modifiable nature of the analyzed factors. In fact, factors such as the type of induction, the position of the mother during delivery, and an increase in the mother’s weight in kilograms in comparison to that at the beginning of pregnancy turned out to be statistically significant with respect to the occurrence of OASIS, so it could be fundamental to concentrate on these factors in daily clinical practice. Until our study, few detailed studies had focused on this aspect [13] and its practical implications.

It is difficult to stratify the risk related to obstetric perineal lacerations during childbirth due to the contribution of different potential risk factors, confounders, and protective factors. Performing an episiotomy is an example, given the current evidence in the literature [14]. Our data are probably related to maternal and/or neonatal biometric factors or other intra-partum dynamics that could be explored in detail in future studies.

However, it is essential that each healthcare operator fully analyzes the relevance of this problem. Therefore, it would be appropriate to conduct further studies on a larger sample, mitigating any research bias to make the samples as homogeneous as possible (e.g., with respect to nulliparity/multiparity), and deeply investigate the real rate of use of known preventive maneuvers in the delivery room [15,16,17].

## 5. Conclusions

OASIS influence women’s post-partum quality of life. It is important for doctors and midwives to collaborate to diminish their incidence and possible risk factors.

Multidisciplinary collaboration and teamwork can overcome the barriers that limit the effectiveness of diagnostic and therapeutic practices, making clinical care easier and more efficient for healthcare workers. New information about OASIS, particularly about Western incidence and possible risk factors, could point clinical attention in the direction of modifiable elements (i.e., BMI and type of induction) useful for preventing and stratifying women at greater risk.

Considering these findings, we can work together to improve the quality of obstetric care by identifying more vulnerable patients and minimizing the risk of severe perineal lacerations during childbirth.

## Figures and Tables

**Table 1:** Distribution of maternal characteristics overall and according to case or control status.

Maternal Characteristics	Cases N = 116	Controls N = 348	Total N = 464	Chi Square *p*-Value
	N (%)	N (%)	N (%)	
Maternal age				
<35 years	89 (76.7)	252 (72.4)	341 (73.5)	0.3624
≥35 years	27 (23.3)	96 (27.6)	123 (26.5)	
Ethnicity				
Caucasian	86 (75.4)	241 (70.9)	327 (72.0)	0.3483
Not Caucasian	28 (24.6)	99 (29.1)	127 (27.1)	
BMI kg/m^2^, mean (SD)	23.84 (4.6)	23.70 (4.6)	23.7 (4.6)	0.7802 ^
Smoker				
Yes	2 (1.7)	9 (2.6)	11 (2.37)	0.7387 *
No	114 (98.3)	339 (97.4)	453 (98.3)	
Pathologies				
Yes	15 (13.4)	60 (17.5)	75 (16.5)	0.3099
No	97 (86.6)	283 (82.5)	380 (83.5)	
Miscarriages				
0	96 (82.8)	249 (71.6)	345 (74.4)	0.0436
1	16 (13.8)	70 (20.1)	86 (18.5)	
>1	4 (3.4)	29 (8.3)	33 (7.1)	
Previous cesarean section				
Yes	12 (10.3)	10 (2.9)	22 (4.7)	0.0010
No	104 (89.7)	338 (97.1)	442 (95.3)	
Assisted-reproduction pregnancy				
Yes	6 (5.2)	2 (0.6)	8 (1.7)	0.0039 *
No	110 (94.8)	346 (99.4)	456 (98.3)	
Nulliparity				
Yes	101 (89.1)	121 (34.8)	222 (47.8)	<0.0001
No	15 (12.9)	277 (65.2)	242 (52.2)	

* Fisher’s exact test, ^ t test.

**Table 2:** Distribution of pregnancy and delivery characteristics overall and according to case or control status.

Pregnancy and Delivery Variables	Cases N = 116	Controls N = 348	Total N = 464	Chi Square *p*-Value
	N (%)	N (%)	N (%)	
Diabetes				
Yes	10 (8.6)	38 (10.9)	48 (10.3)	0.4814
No	106 (91.4)	310 (89.1)	416 (89.7)	
Weight gain				
<10 kg	29 (25.0)	122 (35.1)	151 (32.5)	0.0863
10–15 kg	66 (56.9)	182 (52.3)	248 (53.5)	
>15 kg	21 (18.1)	44 (12.6)	65 (14.0)	
Birthing classes				
Yes	49 (42.2)	126 (36.3)	175 (37.8)	0.2541
No	67 (57.8)	221 (63.7)	288 (62.2)	
Induction				
Yes	40 (34.5)	79 (22.7)	119 (25.7)	0.0119
No	76 (65.5)	269 (77.3)	354 (74.3)	
Type of induction				
Prostaglandins	30 (75.0)	67 (84.8)	97 (81.5)	0.1928
Other methods	10 (25.0)	12 (15.2)	22 (18.5)	
Labor augmentation				
Yes	24 (20.7)	23 (6.6)	47 (10.1)	<0.0001
No	92 (79.3)	325 (93.4)	417 (89.9)	
Episiotomy				
Yes	14 (12.1)	63 (18.1)	77 (16.6)	0.1303
No	102 (87.9)	285 (81.9)	387 (83.4)	
Gestational age at birth (days), median (Q1–Q3)	280.5 (276.0–286.5)	276.0 (270.0–282.0)	278.0 (272.0–283.0)	<0.0001 §
Post-term pregnancy (>40 ws)				
Yes	57 (49.1)	107 (30.8)	164 (35.3)	0.0003
No	59 (50.9)	241 (69.2)	300 (64.7)	
Preterm delivery				
Yes	1 (0.9)	10 (2.9)	11(2.4)	0.3057 *
No	115 (99.1)	338 (97.1)	453 (97.7)	
Epidural analgesia				
Yes	43 (37.1)	60 (17.2)	103 (22.2)	<0.0001
No	73 (62.9)	288 (82.8)	361 (77.8)	
Position at birth				
Supine	16 (13.8)	100 (28.7)	116 (25.0)	<0.0001
Vertical	20 (17.2)	55 (15.8)	75 (16.2)	
Lithotomy	20 (17.2)	14 (4.0)	34 (7.3)	
Squatting	38 (32.8)	55 (15.8)	93 (20.0)	
Sitting/semi-sitting	7 (6.0)	37 (10.6)	44 (9.5)	
Standing	2 (1.7)	8 (2.3)	10 (2.2)	
Stool	6 (5.3)	9 (2.6)	15 (3.2)	
Lateral	7 (6.0)	42 (12.1)	49 (10.6)	
All fours	0 (0.0)	28 (8.1)	28 (6.0)	
Application of an obstetric suction cup				
Yes	14 (12.0)	17 (4.9)	31 (6.7)	0.0073
No	102 (88.0)	331 (95.1)	433 (93.3)	
Duration of first stage of labor (minutes), median (Q1–Q3)	167.5 (90.0–227.5)	105.0 (60.0–180.0)	120.0 (60.0–195.0)	<0.0001 §
Duration of active phase of labor (minutes), median (Q1–Q3)	50.0 (26.5–81.5)	20.0 (11.0–42.0)	25.0 (13.0–52.0)	<0.0001 §
Midwife’s work experience				
<5 years	18 (15.5)	101 (29.1)	119 (25.7)	0.0037
≥5 years	98 (84.5)	246 (70.9)	344 (74.3)	

* Fisher exact test. § Mann–Whitney test.

**Table 3:** Distribution of newborns’ characteristics overall and according to case or control status.

Newborns’ Variables	Cases N = 116	Controls N = 348	Total N = 464	*p*-Value
	N (%)	N (%)	N (%)	
Fetal head position **:				
LOA	66 (56.9)	221 (63.5)	287 (61.9)	0.3503 *
ROA	44 (37.9)	116 (33.3)	160 (34.5)	
LOP	1 (0.9)	4 (1.2)	5 (1.1)	
ROP	5 (4.3)	7 (2.0)	12 (2.5)	
Fetal birthweight gr, mean (SD)	3489.35 (361.5)	3216.84 (514.9)	3284.97 (495.1)	<0.0001 ^
Fetal head circumference cm, mean (SD)	34.41 (1.3)	33.59 (1.6)	33.80 (1.6)	<0.0001 ^

* Fisher’s exact test; ^ t test; ** LOA and ROA (left and right occiput anterior) and LOP and ROP (left and right occiput posterior); gr = grams; cm = centimeters.

**Table 4:** Relative risk (RR) and corresponding 95% confidence intervals (95%CIs) for the association between maternal, pregnancy, delivery, and newborn characteristics and the risk of OASIs.

Maternal Variables	RR (95%CI)	aRR (95%CI)
Maternal age (≥35 vs. <35)	0.84 (0.58–1.23)	
Ethnicity (Caucasian vs. non-Caucasian)	1.19 (0.82–1.73)	
BMI (increase of 1 point)	1.00 (0.97–1.04)	
Smoking (yes vs. no)	0.72 (0.20–2.56)	
Pathologies (yes vs. no)	0.78 (0.48–1.27)	
Miscarriages:		
1 vs. 0	0.67 (0.42–1.07)	
>1 vs. 0	0.44 (0.17–1.11)	
Previous caesarean section (yes vs. no)	2.32 (1.53–3.52)	1.51 (0.85–2.68)
Assisted-reproduction pregnancy (yes vs. no)	3.11 (2.02–4.79)	
Nulliparity (yes vs. no)	7.34 (4.4–12.23)	
Diabetes (yes vs. no)	0.82 (0.46–1.45)	
Weight gain:		
10–15 vs. <10 kg	1.39 (0.94–2.04)	
>15 vs. <10 kg	1.68 (1.04–2.72)	
Attended birthing classes (yes vs. no)	1.20 (0.88–1.65)	
Pregnancy, delivery, and newborn variables		
Induction (yes vs. no)	1.53 (1.11–2.10)	
Labor augmentation (yes vs. no)	2.31 (1.66–3.23)	
Episiotomy (yes vs. no)	0.69 (0.42–1.14)	0.34 (0.20–0.59)
Gestational age at birth (increase of 1 day)	1.05 (1.03–1.07)	1.03 (1.01–1.05)
Post-term pregnancy (>40 ws) (yes vs. no)	1.77 (1.3–2.41)	
Preterm delivery (yes vs. no)	0.36 (0.05–2.34)	
Epidural analgesia (yes vs. no)	2.06 (1.52–2.81)	
Use of an obstetric suction cup (yes vs. no)	1.92 (1.26–2.93)	2.14 (1.38–3.31)
First stage of labor (increase of 10 min)	1.03 (1.02–1.04)	1.02 (1.00–1.03)
Second stage of labor (increase of 10 min)	1.13 (1.09–1.17)	1.12 (1.08–1.16)
Fetal birthweight (increase of 1 hg)	1.10 (1.07–1.14)	
Fetal head circumference (increase of 1 cm)	1.35 (1.21–1.50)	1.21 (1.08–1.35)

aRR: adjusted relative risk, cm: centimeters, and ws: weeks.

## Data Availability

Data are contained within the article.
